# Radiofrequency ablation in the treatment of atypical cartilaginous tumours in the long bones: lessons learned from our experience

**DOI:** 10.1007/s00256-018-3078-2

**Published:** 2018-09-29

**Authors:** Edwin F. Dierselhuis, Jelle Overbosch, Thomas C. Kwee, Albert J. H. Suurmeijer, Joris J. W. Ploegmakers, Martin Stevens, Paul C. Jutte

**Affiliations:** 1 Department of Orthopaedics, Radboudumc, Postbus 9101, 6500 HB Nijmegen, the Netherlands; 2 Department of Radiology, University of Groningen, University Medical Center Groningen, Groningen, The Netherlands; 3 Department of Pathology and Medical Biology, University of Groningen, University Medical Center Groningen, Groningen, The Netherlands; 4 Department of Orthopaedics, University of Groningen, University Medical Center Groningen, Groningen, The Netherlands

**Keywords:** Atypical cartilaginous tumours, Low-grade chondrosarcoma, Radiofrequency ablation, Minimally invasive

## Abstract

**Background:**

Surgery is the cornerstone of treatment of symptomatic cartilaginous neoplasms. We previously studied the application of radiofrequency ablation of atypical cartilaginous tumours in the long bones. The purpose of the present study was to investigate the additional effect of placing multiple needles and a longer procedure duration on the proportion of completely ablated tumours. Post-ablation MRI findings and the occurrence of complications were also assessed.

**Methods:**

We prospectively included 24 patients with atypical cartilaginous tumours in the long bones. Patients underwent CT-guided radiofrequency ablation followed by curettage with adjuvant phenolisation 3 months later, retrieving material assessed for viable tumour. Before curettage, gadolinium-enhanced MRI was performed to check for residual tumour. The occurrence of complications was noted.

**Results:**

Complete tumour ablation was achieved in 17 out of 24 patients (71%). Complete ablation was achieved in 5 of the 6 cases (83%) when multiple needles were used in tumours ≥30 mm. There was incomplete ablation in 8% of patients. Post-ablation gadolinium-enhanced MRI findings agreed with the histological results in 17 out of 23 cases and there was a negative predictive value of 83%. One patient suffered a fracture after radiofrequency ablation.

**Conclusion:**

Radiofrequency ablation could be an alternative to curettage when treating atypical cartilaginous tumours in the long bones. It was shown that multiple needle placement in addition to longer duration of the ablation procedure is an effective measure in achieving complete ablation in tumours ≥30 mm. Gadolinium-enhanced MRI has a negative predictive value of 83% and could guide post-ablation follow-up.

## Introduction

Atypical cartilaginous tumours (ACTs), also known as chondrosarcoma grade I, are bone tumours of borderline or low malignant potential [1]. These lesions increasingly present as a coincidental finding, when patients are evaluated for other bone- or joint-related conditions [2–4]. ACTs are a type of cartilage-forming neoplasm, but unlike higher-grade tumours they do not generally metastasise (<2%) and show excellent survival rates, with <3% local recurrences [5]. Correct diagnosis in the past has been deemed rather difficult, as histological or radiological features alone are not always conclusive [6]. Consequently, tumour upgrading was seen in some cases of local recurrence. For this reason, wide resection of the bone and surrounding tissue used to be recommended sometimes even as primary treatment to avoid this risk [7]. However, recent literature shows that atypical cartilaginous tumours in the appendicular skeleton can be safely treated by curettage with adjuvant phenolisation or cryotherapy, provided that local recurrence rates are low (0–7.7%) and have no negative effect on patient survival [5, 6, 8–16]. Application of this surgical technique has led to an improvement in functional results, although complications such as fracturing may still occur in up to 13% of cases [6, 8–13]. In this context, the tumour biology should be weighed against the morbidity of intralesional surgery. For this reason, some favour a conservative approach, but data are scarce and only retrospective [17]. Minimally invasive treatment may be an alternative, with the advantage of local control, but largely without the burdens of conventional surgery.

In orthopaedic oncology there is increasing interest in the thermal ablation of bone tumours. Radiofrequency ablation (RFA) is a minimally invasive and highly accurate treatment tool. Thermal ablation by RFA has become the gold standard for treatment of certain benign bone tumours (i.e. osteoid osteomas) and can be advantageous in the treatment of skeletal metastases or solid organ tumours (i.e., renal cell carcinoma and hepatocellular carcinoma) [18–21].

In a previous proof-of-principle study by our group, ablation efficacy of RFA in ACTs was assessed by MRI and subsequent histological examination of ablated tumour tissue. Occurrence of complications and short-term functional outcome were also assessed [22]. Complete necrosis was achieved in 45% of patients, whereby size and localisation of the tumour were the main predictors of failure. This result was promising but not satisfactory. Tumours more than 30 mm in diameter were prone to incomplete ablation. The significance of the “heat sink” effect could not be demonstrated. We therefore altered the protocol so that tumours ≥30 mm were to be ablated using multiple needle placement. We also increased the amount of energy delivered with more ablation cycles. The purpose of the current study is to report on the effect of these measures on the proportion of completely ablated tumours, the correlation with post-ablation MRI findings and the occurrence of complications.

## Materials and methods

### Design

**Fig. 1:**
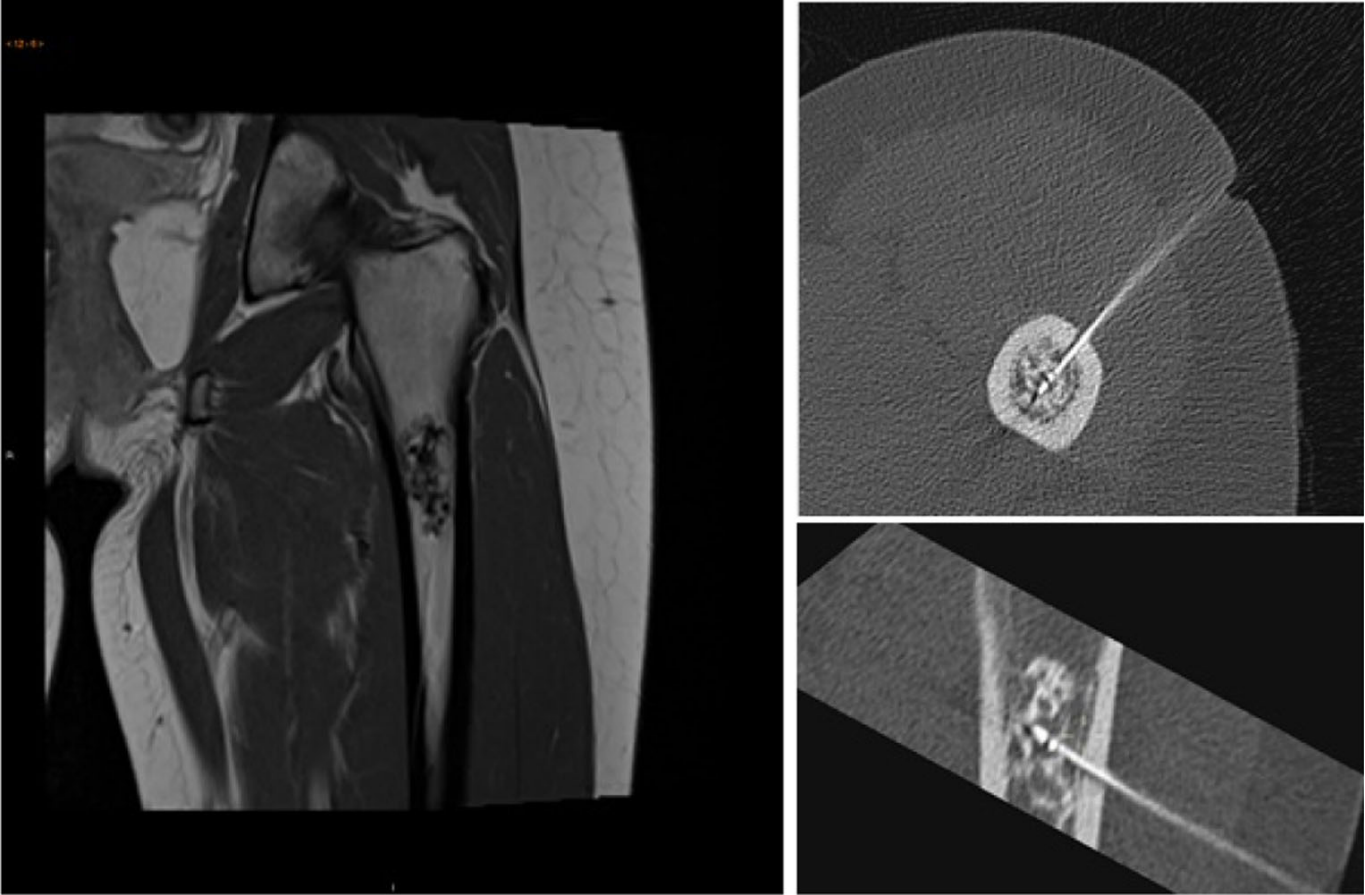
**a** Representative MRI of an atypical cartilaginous tumour (ACT) in the proximal femur. **b** Transverse and **c** coronal images of CT-guided radiofrequency ablation (RFA) of the same tumour

A prospective cohort study was conducted among patients with ACT in the long bones. Inclusion criteria were: patients aged ≥18 with a diagnosis of ACT in the long bones on MRI (e.g. septonodular gadolinium enhancement, no or limited endosteal scalloping, no perilesional oedema), who opted for surgical intervention (Fig. 1). Other indications for surgery were growth of the tumour over time and/or persistent pain at the tumour site. Tumour size was limited to 50 mm maximum diameter in any plane. Tumours were not included if located in the hand, foot, pelvis or axial skeleton. Other exclusion criteria were the presence of cognitive impairments, cortical breakthrough and previous treatment of the same lesion. Written informed consent was obtained from all participants. The study was approved by the medical ethical review committee of our hospital (METc no. M09.077334). All procedures performed in studies involving human participants complied with the ethical standards of the institutional and/or national research committee and with the 1964 Declaration of Helsinki and its subsequent amendments or comparable ethical standards.

### Methods: ablation and surgical technique

**Fig. 2:**
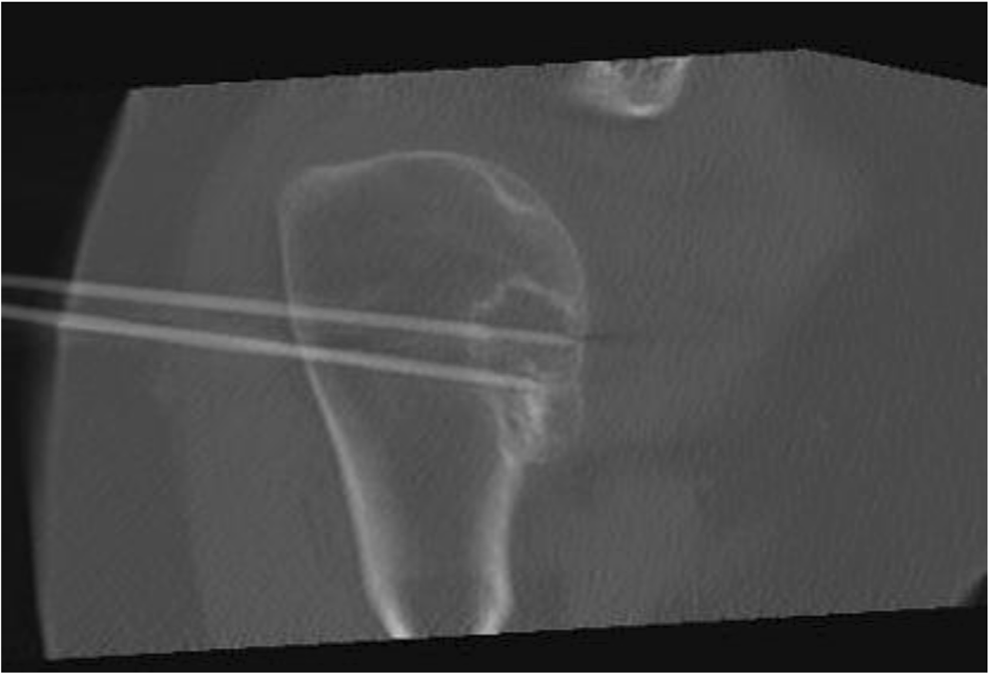
Radiofrequency ablation procedure of an ACT in the proximal humerus with two-needle placement

A CT-guided biopsy under general or spinal anaesthesia, followed by RFA in the same session, was conducted as previously reported [22]. Three months later, gadolinium-enhanced magnetic resonance imaging (Gd-MRI) was performed to assess for completeness of tumour ablation, followed within 4 weeks by curettage and adjuvant phenolisation. The ablation session was performed by one of our consultant musculoskeletal interventional radiologists using a Soloist™ Single Needle Electrode (Boston Scientific, Natick, MA, USA; Fig. 1). The session started with 2 W, adding 1 W every minute, and ended automatically when the needle reached its point of roll-off due to highly elevated impedance of the ablated tissue. Multiple needle placement was applied when incomplete ablation was anticipated in tumours measuring ≥30 mm (Fig. 2). Material obtained during biopsy was examined by a pathologist with special expertise in bone and soft-tissue tumours (A.S). Patients were discharged from the hospital on the same day.

**Fig. 3:**
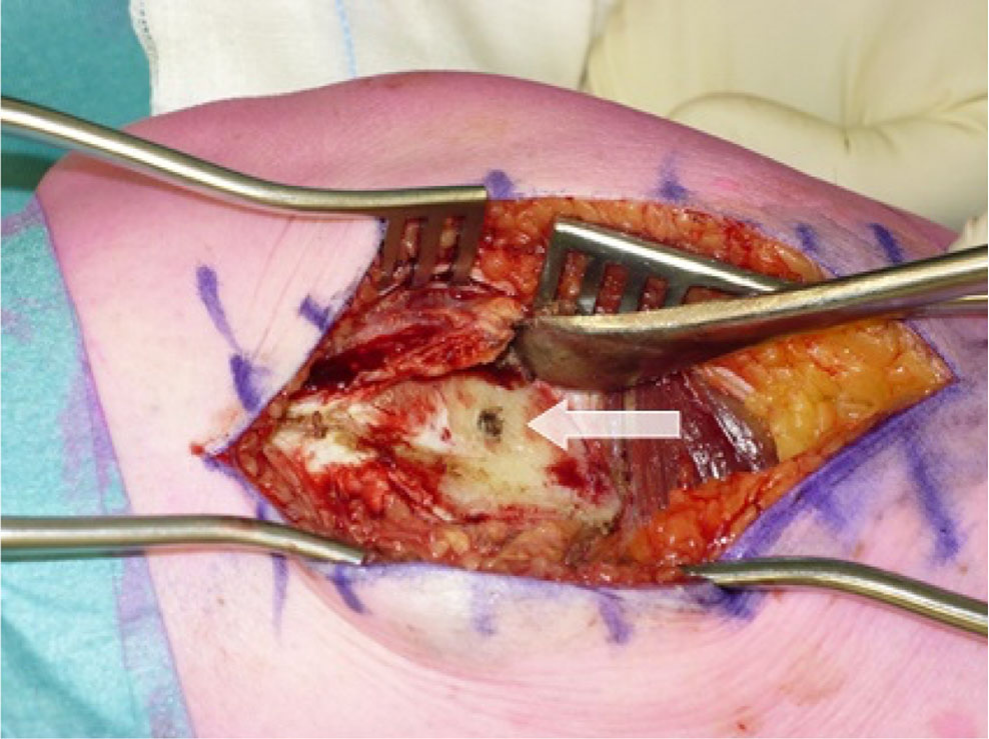
Exposure of the bone to reach the tumour through a cortical window that has to be created. Note the scar from the previous insertion of an RFA needle (*white arrow*)

Curettage was performed in accordance with our usual procedure: a cortical window was created and the lesion was curetted (Fig. 3). After removal of the tumour, phenolisation of the cavity was carried out for 2 min, followed by ethanol washout and saline rinsing. Polymethylmethacrylate (PMMA) was used to fill the defect in all cases. The retrieved material was sent to pathology for histological confirmation of ACT and assessment of the proportion of necrotic tumour tissue. All surgical procedures were performed by one of two orthopaedic oncologists (P.J. and J.P.).

### Pathology

The endpoint was the success rate expressed as a percentage of patients in whom tumour ablation was complete on histology. Reaching 100% cell necrosis was regarded as a pR0 response. Subtotal (95–99%) or incomplete (<95%) tumour eradication were considered pR1 and pR2 respectively. Correlation with post-ablation Gd-MRI findings was noted, in addition to the occurrence of complications.

### Radiology

Measurements of tumour size (largest diameter in any plane) were based on 4-mm slice MR images and 1.5-mm slice CTs. Imaging was also analysed using a grading system that included three categories: no signs of residual tumour (rR0), little or doubtful gadolinium uptake at the tumour border (rR1), and clear residual tumour outside the ablation zone (rR2). Needle positioning was assessed retrospectively. All post-RFA Gd-MRI were graded by a musculoskeletal radiologist (J.O.), who was blinded to the histological results.

### Statistical analysis

Mean and range of values were noted for all variables. SPSS version 22.0 software (IBM-SPSS, Armonk, NY, USA) was used for all statistical testing. If applicable, a univariate analysis was undertaken using Student’s *t* test for normally distributed values and the Mann–Whitney *U* test for non-parametric data; a *p* value <0.05 was considered to be statistically significant.

## Results

### Demographics

In total, 24 patients were included, with a mean age of 51.1 years (range 31–75). The femur was affected most (*n* = 16), followed by the humerus (*n* = 5) and tibia (*n* = 3). Mean tumour size was 28.3 mm (range 15–43). Six patients received multiple needle placement, all in tumours measuring ≥29 mm. The RFA procedure took on average 23.6 min (range 12–37; Table 1). In one patient, a Gd-MRI after RFA was not made, as a fracture occurred before the planned date of the scan. It was a low-energy fracture, 7 weeks after the index ablation procedure. Curettage was performed, followed by mini-open plate fixation without reduction. A non-union developed that needed second surgery with reduction, bone graft and plate fixation. The fracture healed well. This patient was still included for histological analysis purposes.

**Table 1:** Patient characteristics and outcome

Case number	Age (years)	Sex	Location	Diameter (mm)	Ablation duration (min)	Needles	Histological response	Radiological response	Complications
1	56	Female	Femur (M)	24	22	Single	Focal residue	Focal uptake	None
2	63	Female	Femur (M)	21	19	Single	Complete necrosis	No uptake	None
3	51	Female	Femur (M)	30	25	Single	Complete necrosis	No uptake	None
4	67	Female	Femur (M)	27	29	Single	Complete necrosis	No uptake	None
5	49	Female	Femur (D)	35	23	Multiple	Complete necrosis	No uptake	None
6	43	Female	Humerus (D)	15	19	Single	Complete necrosis	No uptake	None
7	31	Male	Femur (M)	28	34	Single	Focal residue	No uptake	None
8	52	Female	Humerus (D)	29	14	Multiple	Complete necrosis	No uptake	None
9	46	Female	Femur (M)	31	21	Single	Substantial residue	Substantial uptake	None
10	49	Female	Humerus (D)	29	29	Single	Complete necrosis	Focal uptake	None
11	53	Female	Tibia (D)	24	26	Single	Complete necrosis	No uptake	None
12	48	Female	Femur (D)	22	17	Single	Complete necrosis	No uptake	None
13	63	Female	Femur (D)	29	37	Single	Complete necrosis	–	Fracture
14	48	Male	Femur (D)	21	15	Single	Complete necrosis	No uptake	None
15	58	Female	Femur (M)	24	12	Single	Substantial residue	Focal uptake	None
16	63	Male	Humerus (D)	34	21	Multiple	Complete necrosis	No uptake	None
17	40	Female	Humerus (D)	36	19	Multiple	Complete necrosis	No uptake	None
18	59	Female	Femur (D)	36	14	Multiple	Focal residue	No uptake	None
19	33	Female	Femur (D)	31	31	Single	Complete necrosis	No uptake	None
20	31	Male	Femur (D)	30	21	Single	Focal residue	Focal uptake	None
21	50	Female	Tibia (D)	43	31	Multiple	Complete necrosis	No uptake	None
22	75	Female	Femur (M)	36	35	Single	Complete necrosis	No uptake	None
23	49	Female	Femur (M)	26	27	Single	Focal residue	No uptake	None
24	47	Female	Tibia (D)	19	25	Single	Complete necrosis	No uptake	None

*M* metaphysis, *D* diaphysis

### Proportion of completely ablated tumours

On a histological level, total ablation (pR0) was reached in 17 out of 24 cases (71%). Incomplete ablation (pR2) was present in 2 out of 24 (8%) and subtotal ablation (pR1) in 5 out 24 (21%) cases. In diaphyseal tumours, pR0 response was achieved in 13 out of 15 (87%) cases compared with 4 out of 9 for metaphyseal tumours (*p* = 0.027). Duration of the ablation procedure was 24.4 min (range 14-37) in pR0, 23.6 min (range 14-34) in pR1 and 16.5 min (range 12-21) in pR2 cases (*p* = NS).

### Correspondence with Gd-MRI

Complete ablation was correctly diagnosed as rR0 in 15 out of 16 cases, with the other case judged as rR1; pR1 corresponded with rR1 in 2 out of 5 and rR0 in 3 out of 5 cases respectively. The cases with a pR2 response were considered rR1 (1 out of 2) and rR2 (1 out of 2; Table 2).

**Table 2:** Correlation of Gd-MRI with histological findings

			Gd-MRI		
		No uptake	Focal uptake	Substantial uptake	Total
pathology	Complete necrosis	15	1	0	16
	Focal residue	3	2	0	5
	Substantial residue	0	1	1	2
Total		18	4	1	23

### Needle positioning

**Fig. 4:**
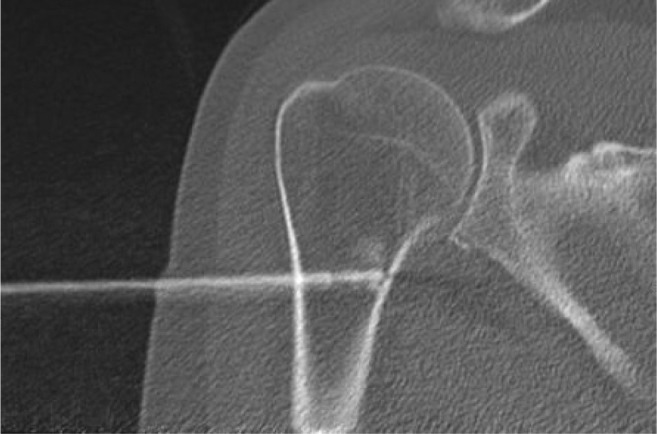
Radiofrequency ablation procedure of ACT in the proximal humerus, with eccentric placement of the needle

For tumours measuring ≤30 mm needle positioning was centric in 11 out of 14 cases, eccentric in 2 cases, and in 1 case multiple needles were applied in a 29-mm diameter tumour. For tumours measuring >30 mm, needle placement was centric in 4 out of 10 cases, multiple needles were applied in 5 out of 10 cases, and in 1 case of a 31-mm tumour the needle was placed eccentrically (Fig. 4). In 1 out of 2 pR2 cases the needle was placed eccentrically (non-significant compared with pR0 and/or pR1 cases). In tumours measuring >30 mm, centric or multiple needle positioning led to pR0 in 7 out of 10 patients and pR1 in 2 out of 10. This group had one pR2 in which the needle was placed eccentrically. When multiple needles were used, complete ablation was achieved in all but 1 case.

## Discussion

We demonstrated in 71% of patients that complete tumour necrosis is achievable using RFA for ACT in the long bones. Implementation of multiple needle placement in larger tumours and longer procedure duration improved ablation effectiveness. After our previous proof-of-principle study, we presented three possible causes of a failed ablation procedure:

1. Number and total ablation time of cycles
2. Tumour size (> 30 mm)
3. Heat sink effect

We slightly adjusted our study ablation technique by delivering more local energy, either by multiple needles or longer ablation duration [22]. We found that in all but 1 case complete ablation was achieved when multiple needles were used. Time is an issue, as temperature rise is a result of conductivity; hence, the longer the procedure takes, the more tissue is heated. Whether the heat sink effect plays a major role in the difference between success rates in metaphyseal and diaphyseal tumours is questionable, but more heat loss to surrounding tissue is plausible in metaphyseal bone if the thinness of the cortex and the higher vascularity of the metaphysis are considered [23].

Although a quantitative comparison with our previous study was not performed, the achieved level of complete necrosis in 71% of the participants was higher than in the proof-of-principle study (45%), with a decrease in evident failures from 30 to 8% in the current study. Based on univariate analysis, diaphyseal tumours are most amenable for RFA treatment, with an 87% success rate. There were 2 patients with substantial viable tissue after ablation. In 1 case, the total ablation time was relatively short (12 min). In the other case, the needle was placed eccentrically and, considering the size (31 mm), there should have been multiple needle placement. Both cases can thus be regarded as technical failures and were not conducted in accordance with our treatment protocol.

Post-ablation Gd-MRI findings corresponded with histological results in 17 out of 23 cases, with 5 cases under-staged (the radiological response was better than the histology) and 1 case over-staged (the radiological response was worse than the histology). Fifteen out of 18 cases were correctly diagnosed as R0 on Gd-MRI (NPV = 83%). We want to stress that both failures (pR2) were seen on Gd-MRI, with one regarded as rR1. There is a chance of a small amount of residual tumour (pR1) being overlooked, but development of local recurrence (out of residue) is very gradual and has no negative effects on patient survival according to the current literature [5]. A recent paper has proposed a classification of MRI response after curettage with a consequent follow-up regime, which in our opinion can be extrapolated to MRI after ablation [24].

Despite the increased efficacy rates compared with our initial proof-of-principle study, there is still room for improvement. We are currently studying needle placement planning, in which ideally an algorithm can be developed using computer modelling and planning with computer-assisted surgery (CAS) to determine and execute optimal needle positioning, especially when multiple probe positions are used. Moreover, a needle that is regulated by temperature sensors instead of impedance could generate a more predictable ablation zone. Real-time imaging of the lesion during ablation would be of great value to monitor the ablation effect, albeit technically demanding. Needles used for thermal ablation are not MRI-compatible, and currently CT cannot detect temperature changes during RFA. Finally, an alternative might be the use of microwave ablation (MWA), as it is less dependent on tissue conductivity than RFA [25].

Our study also has some limitations. Only relatively small lesions were ablated and long-term follow-up after RFA is lacking. Some lesions might arguably have been enchondroma; yet, imaging and biopsy results convinced us of ACT in all cases. In addition, this study was designed as proof-of-principle for whether thermal ablation can treat chondroid tumours and to investigate the reliability of Gd-MRI to check for viable tumour post-ablation. For that reason, curettage served as a control for the effects of RFA at a histological level and assess correspondence of post-RFA histology with Gd-MRI. In the future, RFA will be investigated as a treatment tool instead of curettage, to draw definitive conclusions after adequate follow-up.

To summarise, we have demonstrated that RFA is capable of ablating ACTs in the long bones in 71% of cases, especially diaphyseal tumours. However, long-term follow-up is lacking and future studies should de designed to assess long-term outcome after RFA without subsequent curettage. It should be noted that for many years there has been a dearth of surgical innovations in the treatment of bone tumours, and we believe that the use of local tumour ablation can be a very valuable adjunct to current treatment options. We stress that not all ACTs are candidates for surgery, and yet there is neither a clear consensus on a conservative approach nor clear definitions of indications for surgery [17]. With this study, we have shown that multiple needle placement in addition to longer duration of the ablation procedure is an effective measure for achieving complete tumour ablation in tumours measuring ≥30 mm. Gadolinium-enhanced MRI has a negative predictive value of 83% and could safely guide follow-up after RFA. Future studies should focus on planning, monitoring and further improving ablation efficacy by RFA technique, with adequate follow-up after the ablation procedure.

## References

[1] Hogendoorn PB, Bovee JM, Nielsen GP, Fletcher CDM, Bridge JA, Hogendoorn PCW, Mertens F (2013). Chondrosarcoma (grades I–III), including primary and secondary variants and periosteal chondrosarcoma. World Health Organization classification of tumours of soft tissue and bone.

[2] Hong ED, Carrino JA, Weber KL, Fayad LM (2011). Prevalence of shoulder enchondromas on routine MR imaging. Clin Imaging.

[3] Stomp W, Reijnierse M, Kloppenburg M, NEO study group (2015). Prevalence of cartilaginous tumours as an incidental finding on MRI of the knee. Eur Radiol.

[4] Kransdorf MJ, Peterson JJ, Bancroft LW (2007). MR imaging of the knee: incidental osseous lesions. Radiol Clin N Am.

[5] Hickey M, Farrokhyar F, Deheshi B, Turcotte R, Ghert M (2011). A systematic review and meta-analysis of intralesional versus wide resection for intramedullary grade I chondrosarcoma of the extremities. Ann Surg Oncol.

[6] Leerapun T, Hugate RR, Inwards CY, Scully SP, Sim FH (2007). Surgical management of conventional grade I chondrosarcoma of long bones. Clin Orthop Relat Res.

[7] Eriksson AI, Schiller A, Mankin HJ (1980). The management of chondrosarcoma of bone. Clin Orthop Relat Res.

[8] Aarons C, Potter BK, Adams SC, Pitcher JD, Temple HT (2009). Extended intralesional treatment versus resection of low-grade chondrosarcomas. Clin Orthop Relat Res.

[9] Di Giorgio L, Touloupakis G, Vitullo F, Sodano L, Mastantuono M, Villani C (2011). Intralesional curettage, with phenol and cement as adjuvants, for low-grade intramedullary chondrosarcoma of the long bones. Acta Orthop Belg.

[10] Dierselhuis EF, Gerbers JG, Ploegmakers JJ, Stevens M, Suurmeijer AJ, Jutte PC (2016). Local treatment with adjuvant therapy for central atypical cartilaginous tumors in the long bones: analysis of outcome and complications in one hundred and eight patients with a minimum follow-up of two years. J Bone Joint Surg Am.

[11] Donati D, Colangeli S, Colangeli M, Di Bella C, Bertoni F (2010). Surgical treatment of grade I central chondrosarcoma. Clin Orthop Relat Res.

[12] Hanna SA, Whittingham-Jones P, Sewell MD (2009). Outcome of intralesional curettage for low-grade chondrosarcoma of long bones. Eur J Surg Oncol.

[13] Kim W, Han I, Kim EJ, Kang S, Kim HS (2015). Outcomes of curettage and anhydrous alcohol adjuvant for low-grade chondrosarcoma of long bone. Surg Oncol.

[14] Meftah M, Schult P, Henshaw RM (2013). Long-term results of intralesional curettage and cryosurgery for treatment of low-grade chondrosarcoma. J Bone Joint Surg Am.

[15] van der Geest IC, de Valk MH, de Rooy JW, Pruszczynski M, Veth RP, Schreuder HW (2008). Oncological and functional results of cryosurgical therapy of enchondromas and chondrosarcomas grade 1. J Surg Oncol.

[16] Verdegaal SH, Brouwers HF, van Zwet EW, Hogendoorn PC, Taminiau AH (2012). Low-grade chondrosarcoma of long bones treated with intralesional curettage followed by application of phenol, ethanol, and bone-grafting. J Bone Joint Surg Am.

[17] Deckers C, Schreuder BH, Hannink G, de Rooy JW, van der Geest IC (2016). Radiologic follow-up of untreated enchondroma and atypical cartilaginous tumors in the long bones. J Surg Oncol.

[18] Rosenthal DI, Hornicek FJ, Torriani M, Gebhardt MC, Mankin HJ (2003). Osteoid osteoma: percutaneous treatment with radiofrequency energy. Radiology.

[19] Weis S, Franke A, Mössner J, Jakobsen JC, Schoppmeyer K (2013). Radiofrequency (thermal) ablation versus no intervention or other interventions for hepatocellular carcinoma. Cochrane Database Syst Rev.

[20] Katsanos K, Mailli L, Krokidis M, McGrath A, Sabharwal T, Adam A (2014). Systematic review and meta-analysis of thermal ablation versus surgical nephrectomy for small renal tumours. Cardiovasc Intervent Radiol.

[21] Goetz MP, Callstrom MR, Charboneau JW (2004). Percutaneous image-guided radiofrequency ablation of painful metastases involving bone: a multicenter study. J Clin Oncol.

[22] Dierselhuis EF, van den Eerden PJ, Hoekstra HJ, Bulstra SK, Suurmeijer AJ, Jutte PC. Radiofrequency ablation in the treatment of cartilaginous lesions in the long bones: results of a pilot study. Bone Joint J. 2014;96-B(11):1540–5. 10.1302/0301-620X.96B11.33544 25371471

[23] Hong K, Georgiades C (2010). Radiofrequency ablation: mechanism of action and devices. J Vasc Interv Radiol.

[24] Verdegaal SH, van Rijswijk CS, Brouwers HF (2016). MRI appearances of atypical cartilaginous tumour/grade I chondrosarcoma after treatment by curettage, phenolisation and allografting: recommendations for follow-up. Bone Joint J.

[25] Hinshaw JL, Lubner MG, Ziemlewicz TJ, Lee FT, Brace CL (2014). Percutaneous tumor ablation tools: microwave, radiofrequency, or cryoablation—what should you use and why?. Radiographics.

